# Isolated Trochlear Nerve Palsy Caused by a Cavernous Sinus Dural Arteriovenous Fistula: A Case Report

**DOI:** 10.7759/cureus.96641

**Published:** 2025-11-12

**Authors:** Takashi Hanyu, Osamu Narumi, Daiki Murata, Akihiro Ito, Takaho Murata

**Affiliations:** 1 Neurosurgery, Murata Hospital, Osaka, JPN

**Keywords:** cavernous sinus (cs), cavernous sinus dural arteriovenous fistula, cerebral catheter angiography, diplopia, intracranial dural arteriovenous fistula, trochlear nerve palsy

## Abstract

Cavernous sinus dural arteriovenous fistula (CS-dAVF) usually presents with ocular signs such as conjunctival hyperemia, proptosis, and diplopia. Isolated cranial nerve palsy without ocular signs is uncommon, and isolated trochlear nerve palsy as the sole manifestation is particularly rare.

An octogenarian female presented to our institution with acute diplopia. The Bielschowsky head tilt test revealed her right trochlear nerve palsy. No abnormalities found in other cranial nerves. Magnetic resonance angiography and digital subtraction angiography identified a dural shunt from the right meningohypophyseal trunk to the posterolateral margin of the right cavernous sinus without reflux into cortical veins or the ophthalmic vein, establishing the diagnosis of CS-dAVF.

The long course of the trochlear nerve makes it vulnerable to hemodynamic or mechanical stress. Localized shunt inflow to the lateral compartment can produce selective trochlear nerve palsy without ocular signs. This case indicates that CS-dAVF should be among the differential diagnoses of isolated trochlear nerve palsy. Given the absence of cortical venous reflux, the patient was managed conservatively with observation.

## Introduction

Cavernous sinus dural arteriovenous fistula (CS-dAVF) is an abnormal communication between dural arteries and the cavernous sinus, a venous channel located in the parasellar region. This abnormal shunt alters the normal venous drainage pattern within the cavernous sinus and typically presents with ocular or cranial nerve symptoms such as conjunctival hyperemia, proptosis, diplopia, and pulsatile tinnitus, depending on the direction and extent of venous outflow. However, atypical presentations lacking ocular symptoms and presenting solely with cranial nerve palsies have also been reported [1]. In such cases, involvement of a low-flow shunt to the inferior petrosal sinus has been suggested [1]. When cranial nerve palsies are observed, it is important to include CS-dAVF in the differential diagnosis. However, CS-dAVF is a rare disease, and reports of cases lacking ocular symptoms are particularly limited. We report a case of CS-dAVF presenting with trochlear nerve palsy as the sole symptom, incorporating a review of the literature.

## Case presentation

**Figure 1:**
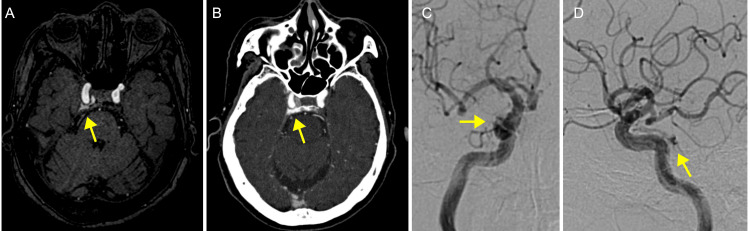
CS-dAVF (A) Magnetic resonance angiography demonstrated an abnormal signal confined to the right cavernous sinus (arrow). (B) Contrast-enhanced computed tomography demonstrated enhancement of the right cavernous sinus (arrow). (C-D) Cerebral angiography (selective right internal carotid angiography) revealed a shunt from the right meningohypophyseal trunk to the posterolateral portion of the right cavernous sinus (arrows).

The patient is an 82-year-old female. She was well until the day before her visit to our institute in June 2025, when she experienced diplopia after flexing her neck to the right side. With right neck flexion, diplopia occurred consistently. Ophthalmologic examination was unremarkable. Neurological examination revealed diplopia when looking downward while sitting facing the examiner. The Bielschowsky head tilt test showed worsening diplopia when tilting the head to the right, leading to a diagnosis of right trochlear nerve palsy. No abnormalities were found in other cranial nerves. No conjunctival injection was observed, and she mentioned no tinnitus or hearing loss. Her medical history included hypertension and dyslipidemia, both being treated. Head magnetic resonance imaging showed no abnormal signal intensity in the brain parenchyma on fluid-attenuated inversion recovery (FLAIR) images, while magnetic resonance angiography (MRA) revealed an abnormal signal confined to the right cavernous sinus (Figure 1A). Computed tomography with contrast material demonstrated enhancement in the right cavernous sinus (Figure 1B). Cerebral angiography revealed a shunt from the right meningohypophyseal trunk to the posterolateral portion of the right cavernous sinus (Figures 1C, 1D). No reflux into the cortical veins or the ophthalmic vein was observed. Based on these findings, a diagnosis of CS-dAVF primarily involving the right cavernous sinus was made. It was classified as Borden type I and Barrow type D. Since symptoms were limited to trochlear nerve palsy and no reflux into cortical veins was present, a decision was made to observe the patient. This decision was also based on the potential risks of new shunt formation or other cranial nerve deficits associated with therapeutic intervention, as well as the possibility of spontaneous improvement.

As of July 2025, conservative observation was continued. Because diplopia due to persistent abducens nerve palsy remained, she was referred to an ophthalmologist at another hospital, where prism glasses and other symptomatic treatments were discussed. She also sought a second opinion from another neurosurgical department for possible curative treatment. Observation and follow-up are ongoing at our institution.

## Discussion

The trochlear nerve originates from the dorsal part of the brainstem, immediately crosses over the superior brainstem tegmentum, and then runs anterolaterally. It then travels anteriorly just beneath the free edge of the tentorium, penetrates the dura mater at the medial side of Meckel's cave, enters the dorsal end of the cavernous sinus, passes through the superior orbital fissure into the orbit, and finally terminates in the superior oblique muscle [2,3]. Thus, the trochlear nerve has a long course and has been noted to be more vulnerable to stimuli compared to other extraocular muscle nerves [4]. This vulnerability is thought to be one reason why trochlear nerve palsy can be evoked by trauma, vascular abnormalities, or compression by a mass lesion.

Given the broad differential diagnosis for trochlear nerve palsy, representative etiologies are summarized below: inflammatory disorders (e.g., neuromyelitis optica [5], multiple sclerosis [6], Tolosa-Hunt syndrome [7]), infections (e.g., Lyme disease [8], tuberculous meningitis [9], herpes zoster ophthalmicus [10]), cerebrovascular lesions (e.g., midbrain infarction [11], posterior cerebral artery aneurysm [12], basilar artery dolichoectasia [13]), and neoplasms (e.g., metastatic melanoma [14]). Rare vascular variants, such as superior cerebellar rete mirabile [15], have also been reported.

The cavernous sinus is a paired venous structure located at the center of the skull base, situated on both sides of the sella turcica and sphenoid sinus. It is divided into four compartments - superior, posterior, inferior, and lateral - based on their spatial relationship with the internal carotid artery. The lateral compartment is located lateral to the anterior to horizontal segments of the internal carotid artery. It is bounded superiorly by the proximal dura mater and inferiorly by the second branch of the trigeminal nerve eminence. Crucial neural structures, including the oculomotor nerve, trochlear nerve, and the first branch of the trigeminal nerve, traverse this compartment [16,17]. In this case, cerebral angiography and head MRA suggested shunt blood flow entering the lateral compartment. Furthermore, given the mechanical vulnerability of the trochlear nerve, localized shunt blood flow to this region may have selectively impaired the trochlear nerve, resulting in isolated superior oblique muscle paralysis.

Reports of CS-dAVF causing isolated trochlear nerve palsy are extremely rare [1,18]. In one case, although typical ocular symptoms like conjunctival hyperemia or exophthalmos were absent, ocular pain was present. In contrast, this case presented no ocular symptoms, including eye pain, and lacked auditory symptoms characteristic of arteriovenous shunt disease, such as tinnitus. The only significant finding was diplopia, representing a highly distinctive clinical presentation. The Bielschowsky head tilt test is useful for diagnosing trochlear nerve palsy and was also helpful in this case. Head imaging studies performed to exclude an organic lesion finally led to the diagnosis of CS-dAVF.

CS-dAVF is a subtype of intracranial dAVF originating from dural arteries branching off the internal and external carotid arteries, with the superior ophthalmic vein and inferior petrosal sinus as its primary venous drainage pathways. Among intracranial dAVFs, lesions associated with cortical venous drainage (CVD) have been reported to have an annual mortality rate of 10.4%, and surgical intervention is recommended as they are considered high-risk lesions [19]. Conversely, in cases without CVD, the incidence of intracranial hemorrhage or non-hemorrhagic neurological deficits is extremely low: 0% over 5.6 years of follow-up and 1.8% over 2.3 years in another report [20,21]. Furthermore, the progression rate from low-risk to high-risk type is reported to be only about 1% per year, making close clinical observation appropriate.

Regarding treatment strategy, surgical intervention, including endovascular treatments such as transvenous embolization, is the standard for CS-dAVFs with CVD. Conversely, conservative management has demonstrated efficacy in low-risk cases without CVD. Reports indicate that intervention carries a significantly higher risk of dAVF-related mortality and permanent complications from stroke in the treated group [22].

In this case, imaging findings showed no CVD. Therefore, the potential for progression to a high-risk lesion was considered low. Considering the prognosis, surgical intervention was deemed inappropriate at this time. Conservative management with careful follow-up was considered the appropriate approach.

## Conclusions

When we encounter a patient with isolated trochlear nerve palsy, CS-dAVF should be included in the differential diagnosis. An imaging workup is warranted to assess for a shunt. If results are inconclusive or suspicion remains high, catheter angiography should be performed to confirm the diagnosis and guide risk-stratified management.
